# Co‐segregation of a homozygous *SMN1* deletion and a heterozygous *PMP22* duplication in a patient

**DOI:** 10.1002/ccr3.645

**Published:** 2016-08-09

**Authors:** Raquel M. Fernández, Ana Peciña, Beatriz Muñoz‐Cabello, Guillermo Antiñolo, Salud Borrego

**Affiliations:** ^1^ Department of Genetics, Reproduction and Fetal MedicineInstitute of Biomedicine of Seville (IBIS)University Hospital Virgen del Rocío/CSIC/University of SevilleSevilleSpain; ^2^ Centre for Biomedical Network Research on Rare Diseases (CIBERER)SevilleSpain; ^3^ Department of PediatricsUniversity Hospital Virgen del RocíoSevilleSpain

**Keywords:** Charcot‐Marie‐Tooth 1A, co‐segregation, double‐trouble cases, genetic analysis, spinal muscular atrophy

## Abstract

Despite co‐segregation of two different genetic neurological disorders within a family is rare, clinicians should take into consideration this possibility in patients presenting with unusual complex phenotypes or with unexpected electrophysiological findings. Here, we report a Spanish 11‐month‐old patient with spinal muscular atrophy type 2 and Charcot‐Marie‐Tooth 1A.

## Introduction

The European Commission on Public Health defines rare diseases as life‐threatening or chronically debilitating diseases, which are of such low prevalence (<1/2000) that special combined efforts are needed to address them. Most rare diseases are genetic, and a great percentage comprises neurological disorders. Co‐segregation of two different genetic neurological disorders within a family is not common, given the low prevalence of this kind of conditions. In the majority of cases, the combined effects of double mutated genes results in more severe phenotypes. Although just a reduced number of those “double‐trouble cases” have been reported, clinical neurologists should take into consideration this possibility in patients presenting with overlapping unusual phenotypes, since a correct and complete diagnosis in the proband is crucial for the genetic counseling and follow‐up in the whole family.

Spinal muscular atrophy (SMA) is an autosomal recessive condition characterized by progressive muscle weakness that results from degeneration and loss of the anterior horn cells in the spinal cord and the brain stem nuclei. Estimated incidence is one in 6000 to one in 10,000 live births and carrier frequency of 1/40–1/60 1. Up to five different subtypes have been described depending on the age of onset: SMA 0 (prenatal onset), SMA I (before 6 months of age, OMIM#253300), SMA II (between 6 and 12 months of age, OMIM#253550), SMA III (after 12 months, in the childhood, OMIM#253400), and SMA IV (with adult onset, OMIM#271150). Around 95% of cases of SMA are caused by homozygous deletions in the *SMN1* gene (5q12.2‐q13.3, OMIM*600354) encoding the SMN (survival motor neuron) protein. A second SMN gene (*SMN2*; 5q13.2, OMIM*601627) has also been identified and contributes to the production of only 10% of the full‐length SMN protein. Therefore, while *SMN1* is undoubtedly the major gene for SMA, disease severity seems to be inversely correlated with the number of copies of the *SMN2* gene, with patients with three or four copies more frequently manifesting SMA3/4, rather than SMA1. In addition, deletions of the *NAIP* gene (5q13.1, OMIM*600355) have also been identified and may play a role in modifying disease severity 1.

Charcot‐Marie‐Tooth neuropathy type 1 (CMT1) is an autosomal dominant demyelinating peripheral neuropathy characterized by distal muscle weakness and atrophy, sensory loss, and slow nerve conduction velocity. It is usually slowly progressive and often associated with *pes cavus* foot deformity and bilateral foot drop. Affected individuals usually become symptomatic between age 5 and 25 years. Up to six clinically indistinguishable subtypes have been described depending on the causing gene. The CMT1A subtype (OMIM#118220) comprises the 70–80% of all CMT1 cases, and its prevalence is approximately 1:3800 to 1:12,500 2, 3, 4, 5. CMT1A is caused by a 1.4‐Mb duplication at 17p11.2 region that includes the *PMP22* gene (OMIM*601097) as the main responsible for the phenotype.

A limited number of families have been reported to be affected by both CMT1A and a second neurologic/neuromuscular condition such as facioscapulohumeral muscular dystrophy 6, X‐linked Charcot‐Marie‐Tooth 7, myotonic muscular dystrophy 7, 8, or Duchenne muscular dystrophy 9.

To date, just one patient has been previously reported with both CMT1A and mild spinal muscular atrophy (SMA 3) 10. Given the known frequencies of CMT and SMA, the coexistence of these two diseases has been estimated as low as 1/18,000,000 10. Here, we report the clinical and electrophysiological findings in another family with a child co‐segregating both disorders and remark the impact of the correct complete diagnosis for all the family members.

## Clinical Report

The proband was a male patient born to a healthy nonrelated couple by spontaneous vaginal delivery at term (39 weeks of gestation). Ultrasound examinations along the whole gestation showed no relevant clinical findings. Birth weight was 3100 g, length was 48 cm, and the cranial perimeter was 34.5 cm. At 11 months of age, a notorious flaccidity and incapability to stand without assistance prompted the family to visit the Neuropediatrics Service at our Hospital. Inspection showed that the child responded adequately to visual and auditory stimuli had a social smile and typical babble, and visual fixation and eye tracking were normal. Spontaneous motility was reduced, mainly at the lower limbs level. Deep tendon reflexes were absent in lower limbs and very weak in upper limbs. Global muscle weakness was noticed and finger tremor was observed. Ability to sit independently had been achieved when placed in a sitting position with a good head control. Twitches of the tongue muscle were not observed during the first inspection, although they were detected in subsequent examinations. These clinical findings led to the suspicion of SMA II, and therefore, additional tests such as genetic analysis of *SMN1*/*SMN2*, EMG, and ENG were requested. An informed consent was obtained from all the members of this family or their legal representatives, for clinical and genetic studies. The studies conformed to the tenets of the Declaration of Helsinki.

**Figure 1:**
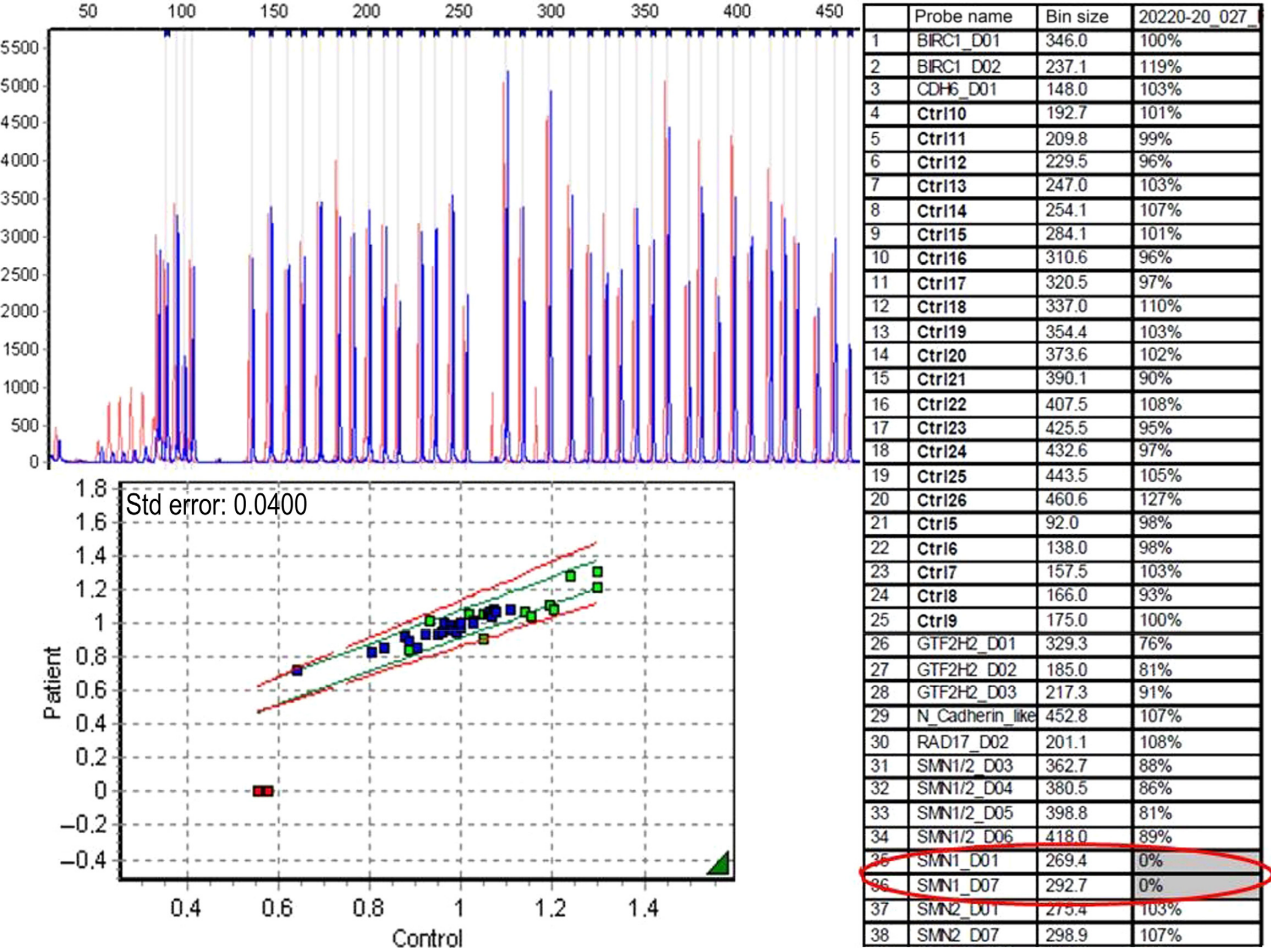
MLPA plot for the analysis of *SMN1* and *SMN2* dosages in our proband (blue) versus a normal control (red). The normal control selected carries two copies for both *SMN1* and *SMN2* genes. Doses for the probes hybridizing with *SMN1* exon 7 (bin size 269.4) and exon 8 (bin size 292.7) are gray shaded, and values of 0% for those probes indicate 0 copies for this gene. Dose values for the probes hybridizing with *SMN2* exon 7 (bin size 275.4) and exon 8 (bin size 298.9) are around 100%, which indicates two copies for this gene.

MLPA methodology 11, was applied for the identification of the number of copies of *SMN1* and *SMN2*, involved in SMA (SALSA P021 MLPA probemix, MRC‐Holland, Amsterdam, the Netherlands). Fragment analyses were performed using the 3730 DNA analyzer (Applied Biosystems, Foster City, CA), and for data analysis, we used GeneMarker v 1.6 (Softgenetics L.L.C). This MLPA analysis in our patient revealed 0 copies of *SMN1* exons 7 and 8, and two copies of *SMN2* exons 7 and 8, which is concordant with the clinical diagnosis of SMA (Fig. 1). In addition, both parents presented with just one copy of *SMN1* and two copies of *SMN2*, confirming their status of asymptomatic SMA carriers.

**Figure 2:**
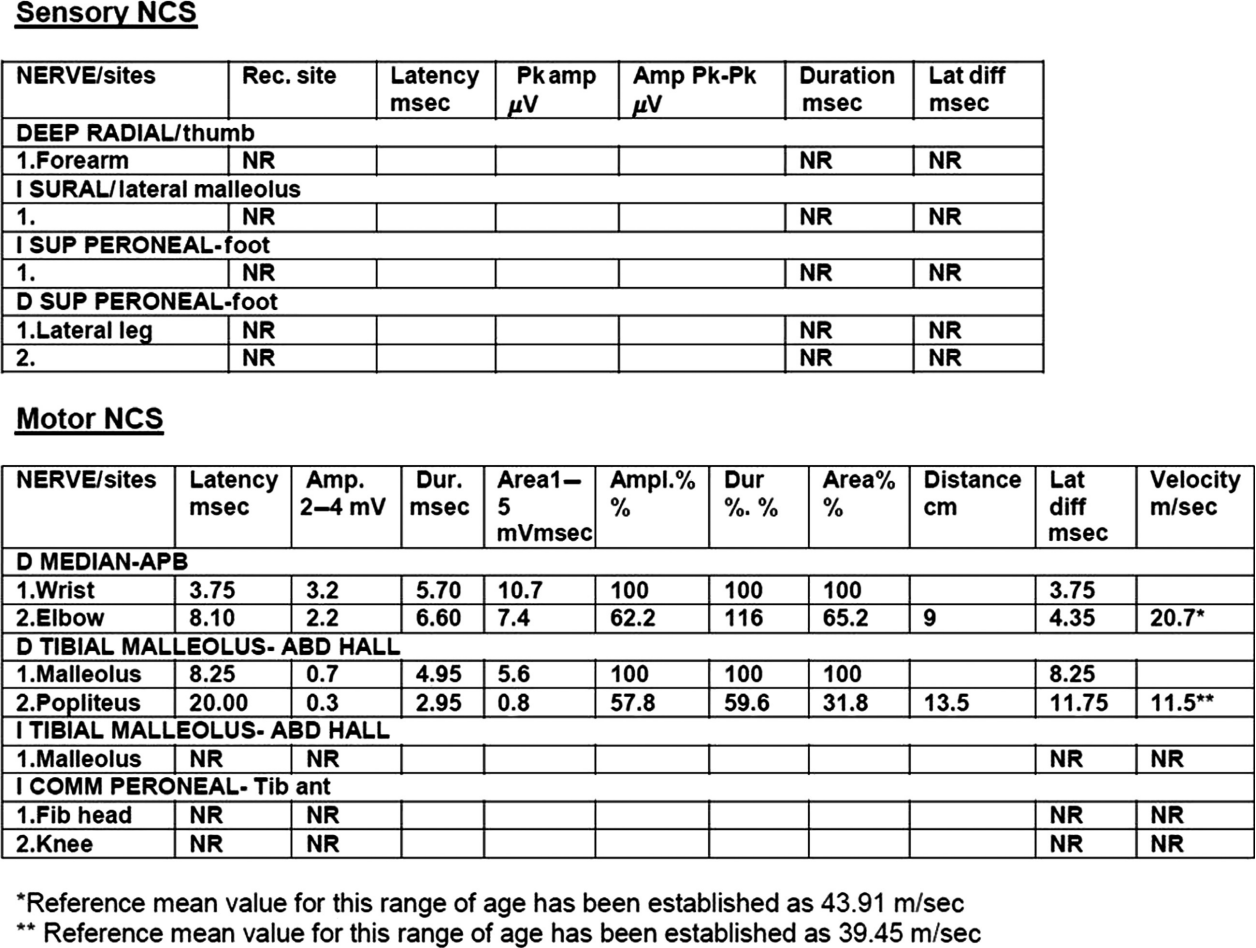
Values registered for both sensory and motor Nerve Conduction Studies (NCS).

**Figure 3:**
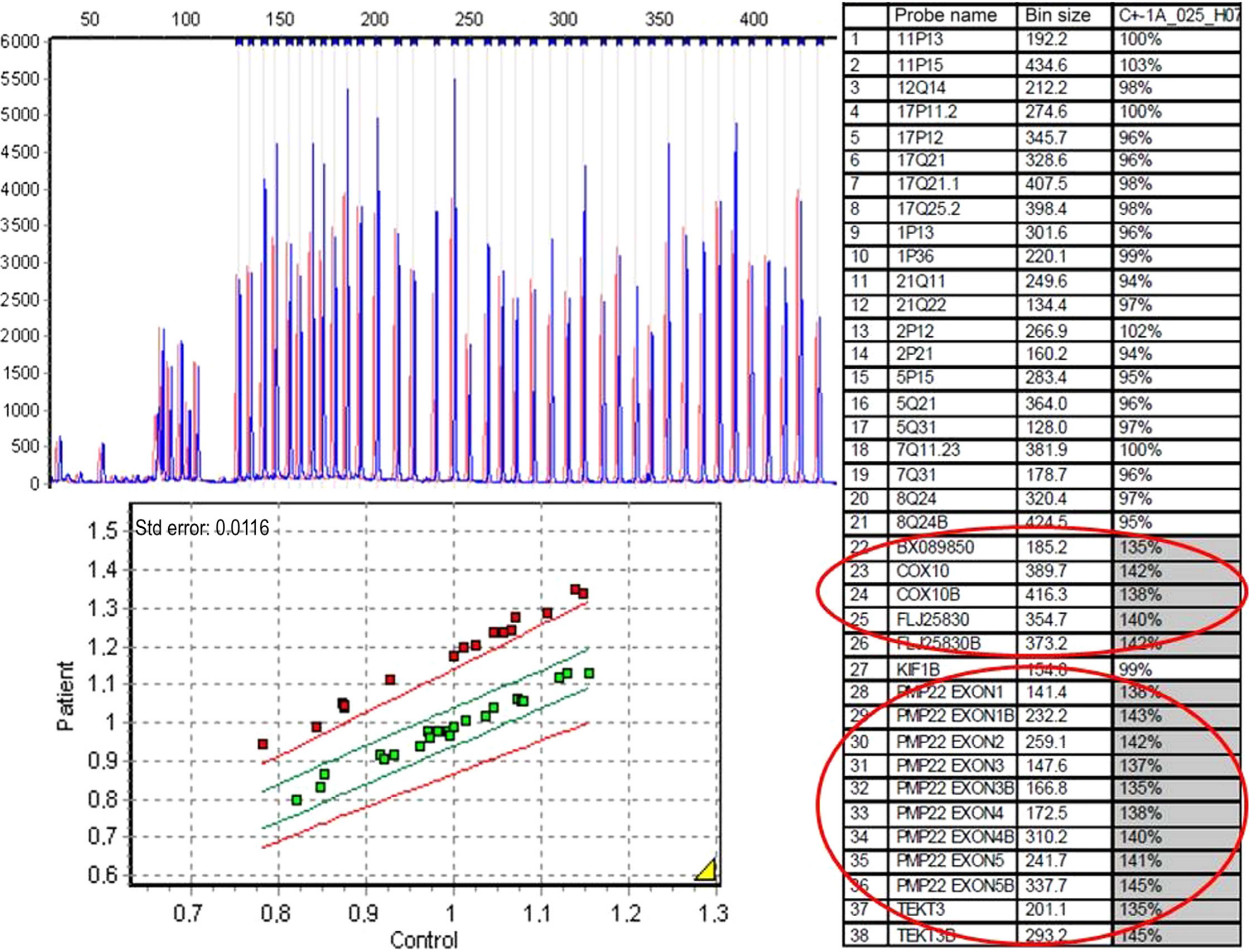
MLPA plot for the analysis of CMT1A critical region in our proband (blue) versus a normal control (red). The normal control selected carries two copies for each of the loci analyzed. Dose values for the probes hybridizing with *COX10, PMP22, FLJ25830,* and *TEKT3,* within the CMT1A critical region, are around 150%, which indicates a duplication of such region.

Unexpectedly, Sensory Nerve Conduction Study (NCS) in the patient showed the absence of response on both lower limbs and on the right upper limb. Moreover, Motor NCS showed slow motor conduction velocities for median and tibial nerves (Fig. 2), when comparing with reference values established for such range of age 12. These findings far from being common in the context of SMA are very typical of a peripheral neuropathy. Thus, a more detailed investigation of the family history was mandatory, and then, the father of the proband mentioned that his brother had been recently diagnosed of CMT1A at the age of 21 years, while he just presented *pes cavus* feet and no other related manifestation at the age of 37 years. Given the remarkable clinical variability of CMT1A in terms of the intensity of clinical features, it was plausible to speculate about the possibility that the father of our proband was carrier of the *PMP22* duplication responsible for CMT1A and had transmitted such mutation to his son. Therefore, genetic analysis of *PMP22* dosage was performed by MLPA (SALSA P033‐B4 CMT1 MLPA probemix, MRC‐Holland, Amsterdam, the Netherlands) in our proband and his father, using the system and the software above described 11. A duplication of the whole gene was observed for both of them, confirming the presence of the molecular cause responsible of CMT1A (Fig. 3).

## Discussion

The presence of atypical features or unexpected findings in the context of neuromuscular diseases should lead us to consider the involvement of more than one genetic event as the cause of the phenotype. The performance of electrodiagnostic tests and DNA analyses is of major interest in the complete elucidation of these unusual complex phenotypes. Sometimes, the combination of two different entities results in a more severe phenotype 9, 13, 14, 15, 16, 17. In other occasions, in contrast, multiple mutations may be associated with milder phenotypes 8, 18. The case here reported represents a different scenario in which an unexpected finding has concluded with the diagnosis of two different entities, although the clinical manifestations correspond only to one of the conditions (SMA). To our knowledge, this is the second case of co‐segregation of SMA‐CMT1A reported so far. In the previous report, the patient was an 8‐year‐old girl affected of CMT1A and SMA type 3 and presented clinical manifestations of both diseases. Our 11‐month‐old patient showed symmetrical proximal muscle weakness typical for the SMA2 form, but none of the typical clinical manifestations of CMT1A. Therefore, no signs would have let the clinician to suspect this neuropathy except the surprising results of the ENG. The observed ENG changes reflecting demyelinating neuropathy prompted the clinician to further investigate the familial medical records and finally to request additional genetic analyses that confirmed the presence of the *PMP22* duplication. The whole findings let to offer genetic analyses to all the members of the family for a presymptomatic study of CMT1A together with the determination of their carrier status for SMA. Moreover, the parents of the patient were informed of the recurrence risks of the two pathologies for future pregnancies and of their reproductive options, including prenatal and preimplantational genetic diagnosis for the two conditions.

This case leads us to think about the possibility that the confluences of different rare genetic conditions might be in fact more common than initially thought, but that they may go unnoticed because a preponderance of the more severe condition and because further genetic studies or additional diagnostic tests are not usually requested once the first mutation that explains the phenotype, at least partially, is identified. Clinicians have to pay attention in these circumstances and to consider to routinely performing additional tests, even in the case that one pathological mutation has already been identified, because a complete and correct diagnosis is crucial for both the genetic and reproductive counseling in the family. Fortunately, nowadays, the increasing development and availability of new powerful tools for DNA analysis such clinical exome sequencing 19 will undoubtedly facilitate the identification of such “double‐trouble” conditions as well as the follow‐up and therapy of the affected patients.

## Conflict of Interest

The authors declared that they have no conflict of interest.
